# The calculation of quality indicators for long term care facilities in 8 countries (SHELTER project)

**DOI:** 10.1186/1472-6963-13-138

**Published:** 2013-04-15

**Authors:** Dinnus HM Frijters, Henriëtte G van der Roest, Iain GI Carpenter, Harriet Finne-Soveri, Jean-Claude Henrard, Angela Chetrit, Jacob Gindin, Roberto Bernabei

**Affiliations:** 1 EMGO Institute for Health and Care Research, VU University Medical Center, Amsterdam, the Netherlands; 2 Centre for Health Service Studies, University of Kent, England, UK; 3 National Institute for Health and Welfare, Helsinki, Finland; 4 Research Unit, Health Environment and Ageing, Sainte Pèrine Hospital APHP and Versailles Saint Quentin University, Paris, France; 5 Center for Standard in Health and Disability, Research Authority, Haifa University, Mount Carmel Haifa, Israel; 6 Centro Medicina dell’Invecchiamento, Università Cattolica del Sacro Cuore, Rome, Italy

**Keywords:** Quality indicators, Long term care facility, SHELTER project

## Abstract

**Background:**

Performance indicators in the long term care sector are important to evaluate the efficiency and quality of care delivery. We are, however, still far from being able to refer to a common set of indicators at the European level.

We therefore demonstrate the calculation of Long Term Care Facility Quality Indicators (LTCFQIs) from data of the European Services and Health for Elderly in Long TERm Care (SHELTER) project. We explain how risk factors are taken into account and show how LTC facilities at facility and country level can be compared on quality of care using thresholds and a Quality Indicator sum measure.

**Methods:**

The indicators of Long Term Care Facility quality of care are calculated based on methods that have been developed in the US. The values of these Quality Indicators (QIs) are risk adjusted on the basis of covariates resulting from logistic regression analysis on each of the QIs. To enhance the comparison of QIs between facilities and countries we have used the method of percentile thresholds and developed a QI sum measure based on percentile outcomes.

**Results:**

In SHELTER data have been collected with the interRAI Long Term Care Facility instrument (interRAI-LTCF). The data came from LTC facilities in 7 European countries and Israel. The unadjusted values of the LTCF Quality Indicators differ considerably between facilities in the 8 countries. After risk adjustment the differences are less, but still considerable. Our QI sum measure facilitates the overall comparison of quality of care between facilities and countries.

**Conclusions:**

With quality indicators based on assessments with the interRAI LTCF instrument quality of care between LTC facilities in and across nations can be adequately compared.

## Background

Quality of care is a complex, multi-dimensional concept. The US Institute of Medicine defines quality of care as “the degree to which health services for individuals and populations increase the likelihood of desired health outcomes which are consistent with current professional knowledge” (http://www.iom.edu). There is interest in the creation of performance indicators that can measure quality by examining the structure, process, and outcomes of care. One method of identifying potentially good and poor professional quality of care is the use of quality indicators, which can be defined as “markers that indicate either the presence or absence of potentially poor care practices or outcomes”. The aim of quality indicators use is therefore to identify the clinical areas that can benefit from improvement of the care process and to define performance of individual care providers [1].

Quality Indicators (QIs) for monitoring quality of care in nursing homes have been developed using assessment data from the widely implemented Resident Assessment Instrument (RAI) for Long Term Care [2-4]. Routine monitoring of these QIs led to QI reports being used for best practice comparison between nursing homes. A study commissioned by the US Centers for Medicare and Medicaid Services (CMS) demonstrated that the items from routine use of the RAI for Long Term Care in US Nursing Homes are reliable and that they can be used for the stimulation of improvement of care and reporting to the general public [5,6]. For most of the QIs some risk adjustment is necessary to allow useful comparison of them between facilities [7,8]. Although the relationship between outcomes and good and bad care practices were not equally strong for all available QIs, 10 QIs had a good enough relationship between identifiable pro-active and responsive care practices. These QIs have been selected by CMS for periodically public reporting at facility level.

A four step approach was used in the CMS commissioned development and validation of QI’s for nursing homes.

1. *Selecting indicators of professional quality of care*. Using large datasets gathered from routine practice, focus groups discussed which assessment items or combination of items might indicate dimensions of quality of care (face validity). QIs then were defined together with the method for calculating numerator and denominator values (construct validity). To be useful the indicators must, in addition, show enough variance between facilities, have high enough prevalence, and show sensitivity to change when care practices change;

2. *Correlating indicators with quality of care*. Experts must agree that high scores (or low scores) on the indicators in a facility or agency correspond to bad (or good) quality of care. This was formalized by research that identified care practices that correlated well with indicator scores pro-actively (i.e. prevent problems) or responsively (i.e. remedy problems);

3. *Identifying person level risk factors.* Factors that legitimately increased or reduced the likelihood of an individual scoring on the indicators were identified by regression analysis of client characteristics as recorded in the assessment items.

4. *Identifying service level bias*. Service level bias (ascertainment bias) manifests itself in two related forms: service/facility admission practice, and staff competence in observation and recording. Nursing homes that admit a relatively large number of clients with some specific indicator problems often continued to score high on these indicators at follow-up, despite risk adjustment. When experts examined the practice of these services/facilities, the quality of care in these indicators areas was not necessarily poor. A Facility Admission Profile (FAP) covariate was defined to resolve this matter [5].

In two papers that came out of the European AgeD in HOme Care (ADHOC) study [9], the calculation of QIs for Home Care was explained and discussed [10,11].

In this paper, similarly, we aim to explain and discuss Quality Indicators for Long Term Care Facilities (LTCFQIs) based on interRAI LTCF assessments [12] and their calculation. To do that we specifically show results of the calculation at country level in the 8 countries participating in the European Services and Health for Elderly in Long TERm care (SHELTER) study [13].

### The list of long term care facility quality indicators

The LTCFQIs shown in this paper were initially commissioned on contract by CMS [5]. The indicators were developed for use with the mandated MDS 2.0 assessments for Nursing Homes. We have ‘translated’ them to be used with the interRAI LTCF instrument [12]. Most could directly be calculated by substituting the MDS 2.0 items with corresponding LTCF items. On occasion some codes in the LTCF items needed to be collapsed to give the same code set as in MDS 2.0. For some of the indicators the conversion of items could not be done or was too complex. The indicators ‘depressed/anxious mood worsening’ and ‘walking performance maintenance/improvement’ were for that reason deleted from the list.

The results of the calculation of the LTCFQI’s have been quarterly reported in the last 6 years to facilities in the Netherlands that used the interRAI LTCF which appears to have had a positive effect on the quality of care in these facilities [14]. On request of these facilities three QI’s were added to the list: ‘Anti-depressant use prevalence’, ‘Influenza vaccination prevalence’ and ‘Depression prevalence’ These QIs were borrowed from the Home Care Quality Indicators set [15] and are included in this study.

Table 1 gives the list of the LTCFQI’s, their name, numerator, denominator exclusions, and risk adjusters.

**Table 1:** Quality indicators for the interRAI LTCF instrument

**# **^**1**^	**Indicator**	**Numerator **^**2**^	**Denominator exclusions **^**3**^	**Risk adjustment**	**FAP** ^**4**^
**Prevalence indicators**					
*Mental functions*					
beh01	Behaviour problem prevalence ^5^	Behaviour symptoms	Comatose.		+
beh02	High risk behaviour problem prevalence	Behaviour symptoms	Comatose.		+
			Low risk behaviour (see beh03)		
beh03	Low risk behaviour problem prevalence	Behaviour symptoms	Comatose.		+
			Dependent in daily decision making or short term memory problem. Schizophrenic or bipolar depression.		
dep01	Depression prevalence	Depression Ration Scale score > = 3			
*Health problems*					
cnt01	Bladder/bowel incontinence prevalence	Frequent or always bladder or bowel incontinent	Comatose, end-stage disease or hospice care.		+
cnt05	High risk bladder/bowel incontinence prevalence	Frequent or always bladder or bowel incontinent	Comatose, end-stage disease or hospice care.		+
			Low risk incontinence (see cnt06)		
cnt06	Low risk bladder/bowel incontinence prevalence	Frequent or always bladder or bowel incontinent	Comatose, end-stage disease or hospice care.		+
			Severely impaired decision making and short term memory problem.		
			Total ADL dependence in bed mobility, transfer toilet or locomotion.		
cnt04	Urinary tract infection	UTI	End-stage disease or hospice care.		
inf01	Infections prevalence	Pneumonia, COPD, septicemia, Sexually transmitted disease, UTI or viral hepatitis	End-stage disease or hospice care.		+
nut01	Feeding tube prevalence		Comatose, end-stage disease or hospice care.	(1) Swallowing problem and stroke	+
bmi0x	Low body mass index prevalence	bmi < = 19	End-stage disease or hospice care.		+
pai01	Inadequate pain management prevalence	Daily moderate or worse pain		(1) Dependent in daily decision making	+
pru01	Pressure ulcer prevalence	PU			+
pru02	High risk pressure ulcer prevalence	PU	Comatose.		+
			Low risk PU (see pru03)		
pru03	Low risk pressure ulcer prevalence	PU	Comatose.		+
			Extensive assistance or more with toilet transfer or bed mobility.		
bur0x	Burns, skin tears or cuts prevalence	Burns, skin tears or cuts			+
*Treatments and procedures*					
soc02	Little/no activity prevalence	Little or no time involved in activities	Comatose, end-stage disease or hospice care.		
drg01	Antipsychotic prevalence	Use of antipsychotic(s)	End-stage disease or hospice care.		+
			Psychotic disorder.		
drg02	High risk antipsychotic prevalence	Use of antipsychotic(s)	End-stage disease or hospice care.		+
			Low risk AP (see drg03)		
drg03	Low risk antipsychotic prevalence	Use of antipsychotic(s)	End-stage disease or hospice care.		+
			Psychotic disorder.		
			Dependent in daily decision making.		
			Short term memory problem.		
			Behaviour symptoms score.		
adp01	Antidepressant prevalence	Use of antidepressant(s)			+
vac01	Influenza vaccination prevalence	Influenza vaccination in last 12 months			
cat02	Indwelling catheter prevalence	Indwelling catheter	End-stage disease or hospice care.	(1) Frequent or always bowel incontinent	
				(2) Stage 3+ PU	
res01	Physical restraints use prevalence	Daily used trunk restraint or chair that prevents rising			
**Incidence indicators**					
*Physical functions*					
adl01	Late-loss ADL decline	Scores as compared to previous scores ^6^ for bed mobility, transfer toilet, eating, toilet use: +1 on at least two of the items or +2 on one item.	Comatose, end-stage disease or hospice care.		
			At previous assessment such a (high/low) score on items that numerator conditions cannot be met. ^7^		
adl02	ADL decline following an improvement	Score on adl-lf ^8^ lower than previous AND score on previous adl-lf higher than at the assessment before previous.	Comatose, end-stage disease or hospice care.		
adl03	ADL improvement	Score on adl-lf lower than previous	Comatose, end-stage disease or hospice care.		
			Person or caregivers do not believe improvement possible.		
mob01	Locomotion worsening	Score on locomotion adl higher than previous	Comatose, end-stage disease or hospice care.	(1) Fell	+
				(2) Extensive assistance eating	
				(3) Extensive assistance toilet use	
fal01	Falls increase	Fell in last 30 days	At previous assessment not a fall in last 30 days	(1) Bedbound	
				(2) Wandering behaviour	
				(3) Unsteady gait and cps > =2	
*Mental functions*					
cog01	Cognitive decline	Score on cps higher than previous	Comatose, end-stage disease or hospice care.	(1) Frequent or always bowel incontinent	+
				(2) Fell in last 30 days	
				(3) Weight loss	
				(4) 76+ years old	
com01	Communication decline	Score on making self understood + ability to understand others higher than previous	Comatose, end-stage disease or hospice care.	(1) Extensive assistance eating	+
				(2) Short term memory	
del01	Delirium new or persistent	Score of 2 on one or more of the delirium items OR a previous score of 2 and now 1 OR now score of 1 and cps < 4	Comatose, end-stage disease or hospice care.		+
beh04	Behaviour problem decline	Sum of behaviour symptoms scores higher than previous	Comatose	(1) Not aphasia	+
			Previous score of 0 on all of the behaviour symptoms	(2) Moderate or severe decision making problem	
*Health problems*					
cnt02	Bowel continence decline	Score on bowel incontinence higher than previous	Comatose, end-stage disease or hospice care.	(1) Short term memory	
				(2) Extensive assistance dressing	
				(3) Frequent or always bladder incontinent	
cnt03	Bladder continence decline	Score on bladder incontinence higher than previous	Comatose, end-stage disease or hospice care.	(1) Short term memory	+
				(2) Extensive assistance dressing	
				(3) Severely impaired decision making	
				(4) Weight loss	
wgt01	Weight loss	5% or more weight loss in last 30 days or 10% or more in last 180 days	End-stage disease or hospice care.	(1) Long term memory	+
			Participant in weight loss program.	(2) Extensive assistance bed mobility	
				(3) Daily or almost daily physically aggressive	
pan01	Pain worsening	Score on pain frequency higher than previous		(1) Dependent in daily decision making	+
pru04	Pressure ulcers worsening	Score on PU stage higher than previous		(1) Extensive assistance with toilet transfer	+
				(2) Fluctuating, precarious, deteriorating condition	
				(3) Extensive assistance with bed mobility	
				(4) Extensive assistance with locomotion	
*Treatments and procedures*					
cat01	New indwelling catheter	Score on indwelling catheter higher than previous	End-stage disease or hospice care.	(1) Frequent or always bowel incontinent	
				(2) Stage 3+ Pressure Ulcer	

^1^ Mnemonics for the quality indicators from megaQI project [5].

^2^ The ADL’s with a 0 – 6 range in the interRAI LTCF have first been recoded into a 0 – 4 range as in RAI/MDS 2.0.

^3^ Condition at target assessment unless stated differently.

^4^ FAP = Facility Admission Profile. FAP-adjustment means correction on the basis of score of the numerator items at admission, if admission occurred in last five quarters.

^5^ All prevalence indicators are calculated at only one, the last, assessment where the person had stayed 30+ days in the facility.

^6^ When an assessment is compared with a previous assessment 6 assessments can be considered, i.e. the target assessment as compared to the previous one; the previous one as compared to the one before that, and so on. It means that in theory a person can be counted 5 times in the calculation of a QI.

^7^ This condition or a similar condition applies to all of the indicators.

^8^ The following scales are used: adl-long form [16], cognitive performance scale [17] and depression rating scale [18].

QI scores are derived from the individual item scores of the interRAI LTCF assessment as indicated in Table 1. They are calculated for the individual person (yes/no/not applicable) and summed per facility or any higher level of aggregation as a numerator/denominator ratio or percentage.

The CMS commissioned study identified risk factors, not necessarily under the control of the facility, that affect the prevalence of some quality indicators [5]. These risk factors are at the level of the individual resident and include differences in various characteristics of the resident, occasionally expressed as a scale value:

• activities of daily living (ADL) ability, measured by the ADL-long form scale – (ADL-lf) [16].

• cognitive function, measured by the Cognitive Performance Scale (CPS) [17].

• depressed mood, measured by the Depression Rate Scale (DRS) [18].

The QI’s are case-mixed corrected by logistical regression analysis, with presence or absence of the QI as the dependent variable and the risk factors as the independent variables. The Facility Admission Profile (FAP) variables are a special kind of risk adjusters in which the values of the QI nominator items at admission are used for risk adjustment.

## Methods

### Design

The SHELTER study has a longitudinal design. Data of residents were collected at baseline, at 6 and at 12 months follow-up.

### Population

The SHELTER data sample consists of Long Term Care Facility residents from 7 European countries, plus Israel [9]. In total 59 private and public LTCFs participated in the study. The number of participating facilities varied widely across the participating countries: 10 in the Czech Republic, 4 in Finland, 6 in France, 9 in Germany, 10 in Italy, 7 in Israel, 4 in the Netherlands, and 9 in England. The aim was to recruit on average 500 residents per country. At baseline data were collected from 4156 residents, at 6 months follow-up data from 3761 residents, and at 12 months follow-up from 2686 residents.

The 24 prevalence indicators, (see Table 1), were to be calculated from the last available assessment of a resident which had been in the facility at that time for 30+ days. The 15 incidence indicators (see Table 1) were to be calculated from the difference between an assessment and the previous assessment of that resident if available.

### Data collection

Assessments were conducted by trained nurses, most of whom worked at the facilities included in the SHELTER study. All were trained in the use of the interRAI-LTCF by experienced trainers in a standardised two day training programme. All trainees received a interRAI-LTCF manual, had access to a Clinical Assessment Protocol manual, and additional training material. On the first day of training the trainees were given an explanation on the interRAI-LTCF and completed a case example from their case load. In the days after they completed an assessment on one or two actual care residents in their facility. On the second day of training those assessments were extensively discussed.

### Analysis

We calculated the LTCFQIs (yes/no/not applicable) for all individuals in the SHELTER dataset [13]. We then entered the risk factors (dependent variable) derived from the CMS study [5] in a stepwise logistic regression analysis for each of the QIs (independent variable) and calculated the Odds Ratios for the risk factors in the SHELTER sample. Since assessment data from the SHELTER study were not necessarily from an initial assessment at admission of the individual to a long term care facility we had no explicit intake data for most of the SHELTER residents. For most residents therefore Facility Admission Profile values were not available.

We compared the unadjusted and risk adjusted individual LTCFQI values by country. Zimmerman showed that for most QIs aggregated facility scores below the 10th percentile scores (indicating ‘better’ care) and above 75th and 90th (indicating ‘worse’ care) are useful for indicating potentially excellent and sub-standard quality of care facilities [3].

We then constructed an *aggregate QI measure* for each country by assigning a score of 1 to every 75th percentile score or above and an extra 1 to every 90th percentile score or above. We used this aggregate score to compare the overall level of deficiency in quality of care of the 59 facilities and the 8 countries in SHELTER. A country or facility will only be compared with other countries or facilities on a LTCFQI, if the actual number of cases with a positive score on the QI or the predicted number of cases with a positive score is 5 or more. A ranking of the countries or facilities is possible by dividing the aggregate QI measure by the number of LTCQIs for which a score was calculated.

Analyses were conducted using SPSS and Microsoft Excel, see Appendix.

Research ethics approval for the SHELTER study was received for all participating countries and specifically from the following ethics committees: METC VUmc Amsterdam, METC Universita Universita Cattolica del Sacro Cuore Rome, METC National Committee, THL Helsinki, METC Hospital Saint Périne Paris, METC Haifa University, Kaplan Medical Center, The Schools Research Committee Ethical Panel, University of Kent Canterbury, METC University of Ulm, Multi-centric Ethics Committee, General Faculty Hospital Prague. Residents were invited to take part in the study and were free to decline participation. Consent was obtained with assurance of data confidentiality.

## Results

The adjusted LTCFQIs by country are shown in Table 2. For each LTCFQI, the best care (lowest LTCFQI score) country is shown light (green) and the worst care (highest LTCFQI score) country is shown dark (red). There is wide variation in the results of some of the LTCFQIs. For example, the LTCFQI “High Risk Behaviour problem prevalence” shows a range from 22% (France) to 61% (England) and the LTCFQI “Physical restraint use prevalence” a range from 1% (England) to 32% (Israel).

**Table 2:** Adjusted long term care quality indicator scores of LTC facilities in 7 European countries and Israel participating in the SHELTER study

	**Czech Republic**	**Finland**	**France**	**Germany**	**Israel**	**Italy**	**Netherlands**	**England**
Behaviour problem prevalence (beh01)	0,37*	0,35	*0,21*	0,34	0,35	0,46	0,46	**0,63**
High risk behaviour problem prevalence (beh02)	0,36	0,39	*0,22*	0,34	0,38	0,48	0,49	**0,61**
Low risk behaviour problem prevalence (beh03)	0,11	0,10	*0,05*	0,07	0,14	0,16	0,11	**0,30**
Depression prevalence (dep01)	0,24	0,39	0,33	*0,24*	0,28	0,36	**0,45**	0,30
Bladder/bowel incontinence prevalence (cnt01)	0,72	**0,91**	0,72	0,75	0,73	*0,63*	0,76	0,81
High risk bladder/bowel incontinence prevalence (cnt05)	0,97	**0,99**	0,97	0,96	0,97	0,96	*0,96*	0,96
Low risk bladder/bowel incontinence prevalence (cnt06)	0,14	**0,46**	0,08	0,21	*0,08*	0,08	0,26	0,15
Urinary tract Infection (cnt04)	0,03	0,01	**0,04**	0,02	0,03	*0,00*	0,03	0,01
Infections prevalence (inf01)	0,07	**0,25**	0,06	0,19	0,12	*0,06*	0,11	0,12
Feeding tube prevalence (nut01)	0,01	*0,00*	0,00	0,03	**0,05**	0,02	0,02	0,01
Low body mass index prevalence (bmi0x )	0,11	0,16	*0,02*	0,05	0,13	**0,30**	0,11	0,30
Inadequate pain management prevalence (pai01)	0,13	0,10	0,05	0,13	*0,04*	0,04	0,14	**0,16**
Pressure ulcer prevalence (pru01)	0,11	*0,06*	0,13	0,10	0,06	**0,13**	0,11	0,08
High risk pressure ulcer prevalence (pru02)	0,17	*0,07*	**0,18**	0,16	0,08	0,17	0,18	0,10
Low risk pressure ulcer prevalence (pru03)	0,03	0,02	0,03	**0,04**	0,02	0,03	0,03	*0,01*
Burns, skin tears or cuts prevalence (bur0x)	0,05	0,03	*0,02*	**0,12**	0,06	0,04	0,06	0,11
Little/no activity prevalence (soc02)	0,57	*0,32*	**0,68**	0,41	0,56	0,46	0,43	0,50
Antipsychotic prevalence (drg01 )	0,30	0,21	0,38	0,36	0,22	0,34	*0,13*	**0,45**
High risk antipsychotic prevalence (drg02 )	**0,78**	*0,41*	0,67	0,64	0,46	0,71	0,42	0,72
Low Risk Antipsychotic prevalence (drg03 )	0,21	0,15	0,21	0,27	0,17	0,22	*0,07*	**0,32**
Antidepressant prevalence (adp01)	0,37	0,30	*0,08*	0,43	0,26	**0,50**	0,22	0,38
Influenza vaccination prevalence (vac01)	0,31	0,08	0,07	**0,35**	0,09	*0,05*	0,11	0,19
Indwelling catheter prevalence (cat02)	0,01	0,02	*0,00*	**0,02**	0,00	0,01	*0,00*	0,01
Physical restraints use prevalence (res01)	0,04	0,13	0,14	0,06	**0,32**	0,21	0,09	*0,01*
Late-loss ADL decline (adl01)	0,20	0,33	0,22	0,22	0,24	0,20	*0,19*	**0,38**
ADL improvement (adl03)	*0,41*	0,26	0,23	0,16	0,24	0,19	**0,14**	0,38
ADL decline following an improvement (adl02)	0,15	0,25	0,16	*0,05*	0,17	0,08	0,09	**0,49**
Locomotion worsening (mob01)	0,26	**0,32**	0,19	0,24	0,24	0,23	*0,16*	0,29
Falls increase (fal01)	0,07	0,11	**0,13**	0,12	0,04	*0,04*	0,11	0,07
Cognitive decline (cog01 )	0,25	0,35	*0,19*	0,36	0,37	0,25	0,30	**0,43**
Communication decline (com01)	0,25	0,38	0,33	0,28	0,30	*0,17*	0,19	**0,39**
Delirium new or persistent (del01)	*0,21*	0,28	0,26	0,40	0,32	0,27	0,36	**0,43**
Behaviour problem decline (beh04)	*0,14*	0,32	0,20	0,24	0,20	0,15	0,28	**0,38**
Bowel continence decline (cnt02)	0,15	0,24	0,23	0,13	0,19	0,18	*0,12*	**0,34**
Bladder continence decline (cnt03)	0,26	**0,39**	0,35	*0,16*	0,18	0,20	0,21	0,29
Weight loss (wgt01)	0,07	*0,03*	0,05	0,06	0,05	0,06	0,07	**0,11**
Pain worsening (pan01)	0,15	0,19	**0,56**	0,13	*0,03*	0,07	0,10	0,19
Pressure ulcers worsening (pru04)	0,05	0,03	**0,08**	0,04	0,04	0,03	0,06	0,04
New indwelling catheter (cat01)	0,01	*0,00*	*0,00*	*0,00*	*0,00*	**0,02**	0,01	0,01

* For each LTCFQI, the best care (lowest LTCFQI score) country is shown in *italics* and the worst care (highest LTCFQI score) country is shown in **boldface**.

Additional file 1: Table S1 shows the *aggregate LTCFQI measure* for each country. The Czech Republic and Israel have by far the lowest summary scores of 5 and 6 (‘best’ quality of care), England the highest score of 33 (‘worst’ quality of care). The aggregate scores of the other countries are in between.

The LTCFQI scores of the 59 facilities can be calculated and presented in the same way. The outcomes for facilities, even in one country, show large differences. For example, the four facilities in Finland that participated in the SHELTER study produced the following results:

• Facility 1: 34 QIs calculated; 7 above the 90th percentile, 8 between the 75th and 90th; ranked 50 out of 59.

• Facility 2: 16 calculated; 2 above the 90th percentile, 1 between the 75th and 90th; ranked 27.

• Facility 3: 36 calculated; 3 and 5; ranked 26.

• Facility 4: 20 calculated; 0 and 1; ranked 2.

## Discussion

In this paper we have explained and discussed the methods for calculating Long Term Care Facility Quality Indicators (LTCFQIs). We used the method to calculate the values of 39 such indicators on assessments from 59 LTC facilities in 7 European countries plus Israel that participated in the Services and Health for Elderly in Long TERm Care (SHELTER) project [13].

Even if the calculations are appropriate, see discussion below, the results of the measurements cannot be considered representative for the eight countries. The recruitment in the SHELTER project was for cost reasons not aimed at representative samples for each country, only a limited number of facilities in each of the countries therefore have participated. The results of individual facilities in a country can be quite different, as shown, as an example, for the four facilities from Finland. Our intention with this paper is to show that professional quality of care can be calculated and compared between locations. To obtain valid comparisons between countries much larger samples of facilities per country need to be included. It is possible, however, that some of the outcomes per country will persist when samples are larger, as is predicted by the experts of LTCF that co-authored this paper.

For the moment, the differences between individual facilities, within and across countries, are probably much more real. If so, what does that imply for an individual facility? This study enables a benchmark on quality of care. Feedback of the results can be used effectively to improve the quality of care in a facility, as has been shown by Boorsma et al. [14] in a RCT study. To achieve this, demands considerable efforts: continued collection of good data, training, routine use of the assessment outcomes in care planning, continued interest by the management of the facilities. The work environment of a Long Term Care Facility is complex with its 24 × 7 hours of care, work shifts, various disciplines with their own manners, interaction with family and volunteers, complex and emotional care demands, budget restraints. Besides, the real effort to improve quality of care needs to be made on the level of the wards, each with its own more or less independent management, work relations, manners and specific groups of residents. Most LTCFQIs, however, cannot be reliably calculated for wards, because of limited nominator and denominator numbers. Additional analysis by management of the facility, therefore, is required to identify problems with quality of care on particular wards.

The validity of the measurements of the LTCFQIs depends first of all on the accuracy of the assessment. In our study the assessors were adequately trained at the start, so that we may assume that the baseline assessment was fine and comparable between facilities in all of the countries [13]. At the time of the second assessment, approximately 6 months later, and the third at twelve months, knowledge may have decreased or increased. Facilities where assessments were completed by nurses that worked in these facilities, may have employed new nurses that possibly have not received the same level of assessment training. Facilities where the assessments have been used in routine care planning, as intended, and where computer output was distributed timely and where the results have been thoroughly discussed, nurses and their care teams have become more experienced and better at recognizing issues that are assessed with the interRAI LTCF instrument. In other facilities, where nothing or less than was intended has been done with the results of the assessment, the opposite can be true. It is even possible that in the latter facilities the second and third assessment have largely been copied from printouts of the baseline assessment. We suspect that some of the incidence LTCFQIs outcomes may have suffered from this. Continued good quality of the assessments needs firm external and internal incentives, as has been shown in the US and Canada where interRAI instruments now have been used on a large scale for some decades [19].

The calculation of QIs presumes that the facilities and their resident population are to a large extent comparable, i.e. in level of care, kind of care, kind of residents, focus of care. This has not necessarily been so in the SHELTER study. The person’s living arrangement at the time of referral varied. In France, 45 percent of the residents came from a rehabilitation hospital. In England and the Czech Republic, most of the residents came from an acute hospital. In Italy, Israel and Germany the majority came from home. The number of residents in various Resource Utilization Groups, and length of stay [not presented in this paper], show that in some facilities (e.g., in Israel) most residents received extensive rehab, or stayed comparatively short (e.g., Czech Republic). Some of this is remedied by the risk adjustment, but likely not all.

In the calculation of the aggregate LTCFQI measure each QI has the same weight and the 75-th and 90-th percentile thresholds are equally important for each QI. These assumptions are obviously flawed. For some quality of care issues a number of QIs are available and for others maybe none. It is known to the authors that efforts are being made by interRAI to develop a more balanced aggregate measure on a subset of the QIs, including adding new QIs to the existing set that look at decline and improvement on particular issues simultaneously. Furthermore, the 75-th and 90-th percentiles are often useful, although not equally meaningful for each QI [3], but this has not been verified for all of the QIs, presented in this paper.

A further remark on the calculation of the QIs concerns the frequency of the assessment and the Facility Admission Profile risk adjustment. In the original research for the development of the QIs [5,6], residents were assessed at least every 3 months. In SHELTER, follow-up assessments were scheduled at 6 and 12 months. This affects the results of the incidence QIs, but probably not a large extent. The FAP values, however, which are used for risk adjustment, could in this study only be calculated when a resident was assessed at entry to the facility at baseline. This was rarely so, since the facilities were selected that had long-stay residents, which by definition have few new entries in a short period of time. And even so, risk adjustment through FAP values is not perfect.

Quality of life as experienced by the resident is as important as the calculation of professional quality of care and should, when possible, be measured and analyzed additionally to evaluate services delivered and prepare for enhanced services.

## Conclusion

To conclude, the LTCQIs are useful measures of professional quality of care in Long Term Care facilities. The indicators are based on the worldwide used interRAI LTCF instrument. When the conditions for measurement are met the indicators appropriately measure quality of care. We have shown how the facilities in the SHELTER study, and to a lesser extent the countries, can be compared with each other with the LTCFQIs.

## Appendix

We calculated the LTCFQIs as follows. We wrote SPSS syntax coding for the indicators, listed in Table 1. We executed this SPSS syntax on the SHELTER data. This generated for each QI an individual value for each resident, SPSS logistic regression analysis output tables for each of the LTCFQIs and risk-adjusted predicted QI values for each SHELTER resident. We transferred per facility (/country) the N of valid QI scores for a QI, the mean value of that QI, and the mean predicted value of the QI score (as derived from the logistic regression analysis), when applicable, to a MS EXCEL application. There we calculated the QI scores per facility (/country). When a QI score needed to be adjusted, we used the formula:

$$\begin{array}{l} \text{Adjusted}\;\text{LTCFQ}I_{x,y}\\ \;=1/(1+\text{exp}(-\text{ln}\left(\text{LTCFQ}I_{x,y}/\left(1-\text{LTCFQ}I_{x,y}\right)\right)\\ \;-\text{ln}\left(\text{predicte}d_{x,y}/\left(1-\text{predicte}d_{x,y}\right)\right)\\ \;+\text{ln}\left(\text{mean}\;\text{LTCFQI}\;\text{score}/\left(1-\text{mean}\;\text{LTCFQI}\;\text{score}\right)\right))), \end{array}$$

where LTCFQI_x,y_ is the score for LTCFQI *x* for facility (/country) *y*, and predicted_x,y_ is the mean value of the predictions of the values of LTCFQI *x* for all individual residents of the facility (/country) *y* as calculated by the SPSS logistic regression analysis.

## Competing interests

The authors declare that they have no competing interests.

## Authors’ contributions

DHMF: substantial contribution to conception and design; analysis and interpretation; main author. GIC: contribution to conception and design; acquisition of data. HGvdR: acquisition of data; analysis and interpretation. RB: contribution to conception and design; acquisition of data. HFS: contribution to conception, acquisition of data; revision of content. JCH: contribution to conception, acquisition of data; interpretation of results; revision of content. AC: acquisition of data; revision of content. JG: acquisition of data; revision of content. All authors read and approved the final version.

## Pre-publication history

The pre-publication history for this paper can be accessed here:

http://www.biomedcentral.com/1472-6963/13/138/prepub

## Supplementary Material

Additional file 1: Table S1 Aggregate summary scores of LTCF quality indicators at the level of the 8 countries participating in SHELTER. Click here for file
