# Supplementary material for: The calculation of quality indicators for long term care facilities in 8 countries (SHELTER project)
Source: BMC Health Serv Res. 2013 Apr 15;13:138. doi: 10.1186/1472-6963-13-138 (PMC3716904; doi:10.1186/1472-6963-13-138)
Supplement: Additional file 1: Table S1 — Aggregate summary scores of LTCF quality indicators at the level of the 8 countries participating in SHELTER. [file 1472-6963-13-138-S1.doc]

Table S1. Aggregate summary scores of LTCF Quality Indicators at the level of the 8 countries participating in SHELTER.

|  | Czech Republic | Finland | France | Germany | Israel | Italy | Nether-lands | England |
| --- | --- | --- | --- | --- | --- | --- | --- | --- |
| Behaviour problem prevalence[1] (beh01) |  |  |  |  |  |  | 1 | 2 |
| High risk behaviour problem prevalence (beh02) |  |  |  |  |  | 1 | 1 | 2 |
| Low risk behaviour problem prevalence (beh03) |  |  |  |  |  |  |  | 2 |
| Depression prevalence (dep01) |  | 1 |  |  |  |  | 2 |  |
| Bladder/bowel incontinence prevalence (cnt01) |  | 2 |  |  |  |  |  | 1 |
| High Risk Bladder/bowel incontinence prevalence (cnt05) |  | 2 |  |  | 1 |  |  |  |
| Low Risk Bladder/bowel incontinence prevalence (cnt06) |  | 2 |  |  |  |  | 1 |  |
| Urinary Tract Infection (cnt04) |  |  | 2 |  |  | N/A | 1 |  |
| Infections prevalence (inf01) |  | 2 |  | 1 |  |  |  |  |
| Feeding tube prevalence (nut01) |  |  |  | 1 | 2 |  |  |  |
| Low Body Mass Index prevalence (bmi0x ) |  |  |  |  |  | 2 |  | 1 |
| Inadequate pain management prevalence (pai01) |  |  |  |  |  |  | 1 | 2 |
| Pressure ulcer prevalence (pru01) |  |  | 1 |  |  | 2 |  |  |
| High Risk Pressure ulcer prevalence (pru02) |  |  | 2 |  |  |  | 1 |  |
| Low Risk Pressure ulcer prevalence (pru03) |  |  |  | 2 |  | 1 |  |  |
| Burns, skin tears or cuts prevalence (bur0x) |  |  |  | 2 |  |  |  | 1 |
| Little/no activity prevalence (soc02) | 1 |  | 2 |  |  |  |  |  |
| Antipsychotic prevalence (drg01 ) |  |  | 1 |  |  |  |  | 2 |
| High Risk Antipsychotic prevalence (drg02 ) | 2 |  |  |  |  |  |  | 1 |
| Low Risk Antipsychotic prevalence (drg03 ) |  |  |  | 1 |  |  |  | 2 |
| Antidepressant prevalence (adp01) |  |  |  | 1 |  | 2 |  |  |
| Influenza vaccination prevalence (vac01) | 1 |  |  | 2 |  |  |  |  |
| Indwelling catheter prevalence (cat02) | N/A^x^ |  | N/A | 2 | N/A | N/A | N/A | N/A |
| Physical restraints use prevalence (res01) |  |  |  |  | 2 | 1 |  |  |
| Late-loss ADL decline (adl01) |  | 1 |  |  |  |  |  | 2 |
| ADL decline following an improvement (adl02) |  | 1 |  |  |  |  |  | 2 |
| ADL improvement (adl03) |  |  |  | 1 |  |  | 2 |  |
| Locomotion worsening (mob01) |  | 2 |  |  |  |  |  | 1 |
| Falls increase (fal01) |  |  | 2 | 1 |  |  |  |  |
| Cognitive decline (cog01 ) |  |  |  |  | 1 |  |  | 2 |
| Communication decline (com01) |  | 1 |  |  |  |  |  | 2 |
| Delirium new or persistent (del01) |  |  |  | 1 |  |  |  | 2 |
| Behaviour problem decline (beh04) |  | 1 |  |  |  |  |  | 2 |
| Bowel continence decline (cnt02) |  | 1 |  |  |  |  |  | 2 |
| Bladder continence decline (cnt03) |  | 2 | 1 |  |  |  |  |  |
| Weight loss (wgt01) |  |  |  |  |  |  | 1 | 2 |
| Pain worsening (pan01) |  | 1 | 2 |  |  |  |  |  |
| Pressure ulcers worsening (pru04) |  |  | 2 |  |  |  | 1 |  |
| New indwelling catheter (cat01) | 1 |  |  |  |  | 2 |  |  |
| *Number of LTCQIs with percentile score* | *38* | *39* | *38* | *39* | *38* | *37* | *38* | *38* |
| **Aggregate summary score** | **5** | **19** | **15** | **15** | **6** | **11** | **12** | **33** |
| **Ranking** | 1 | 7 | 6 | 5 | 2 | 3 | 4 | 8 |

^X^ Not available because the actual number of cases with a positive score and the predicted number with that score are both less than 5
